# Myocardial Perfusion in Rheumatoid Arthritis Patients: Associations with Traditional Risk Factors and Novel Biomarkers

**DOI:** 10.1155/2017/6509754

**Published:** 2017-05-03

**Authors:** Miguel Bernardes, Tiago S. Vieira, Maria João Martins, Raquel Lucas, Lúcia Costa, Jorge G. Pereira, Francisco Ventura, Elisabete Martins

**Affiliations:** ^1^Department of Rheumatology, São João Hospital Center, Porto, Portugal; ^2^Department of Medicine, Faculty of Medicine, University of Porto, Alameda Prof. Hernâni Monteiro, 4200-319 Porto, Portugal; ^3^Department of Nuclear Medicine, São João Hospital Center, Porto, Portugal; ^4^i3s-Instituto de Investigação e Inovação em Saúde, Universidade do Porto, Porto, Portugal; ^5^Department of Biomedicine, Unit of Biochemistry, Faculty of Medicine, University of Porto, Porto, Portugal; ^6^Department of Clinical Epidemiology, Predictive Medicine and Public Health, Faculty of Medicine, University of Porto, Porto, Portugal; ^7^EPI Unit-Institute of Public Health, University of Porto, Porto, Portugal; ^8^Faculty of Medicine, University of Porto, Porto, Portugal; ^9^Department of Cardiology, São João Hospital Center, Porto, Portugal

## Abstract

*Introduction*. Cardiovascular (CV) diseases are a major cause of death in rheumatoid arthritis (RA) patients. Novel biomarkers [B-type natriuretic peptide (BNP); osteoprotegerin (OPG)/receptor activator of nuclear factor-kappa B ligand (RANKL) ratio; and dickkopf-1 (DKK-1)] have been used in CV risk assessment. We analysed, in established RA patients, the presence of silent myocardial ischemia and its association with clinical variables, BNP, and bone and atheroma biomarkers.* Methods*. From a single-center tertiary referral hospital, RA patients asymptomatic for CV disease were submitted to myocardial perfusion scintigraphy (MPS) under adenosine stress and biomarkers measurements. Logistic regression was used to estimate crude odds ratios (OR) and 95% confidence intervals (CI).* Results*. In 189 patients, perfusion defects were frequent (25%) and associated with BNP ≥ 100 pg/mL (OR = 5.68; 95% CI: 2.038–15.830), fourth log OPG/RANKL ratio quartile (OR = 2.88; 95% CI: 1.091–7.622), and DKK-1 ≥ 133 pmol/L (OR = 2.69; 95% CI: 1.058–6.840). Similar associations were confirmed in those with C-reactive protein > or ≤ 3 mg/L. No relationship was found with the majority of traditional CV factors nor with disease variables.* Conclusions*. Our results corroborated the hypothesis that MPS could reveal subclinical CV dysfunction, supported the utility of BNP measurements as a screening tool, and put in perspective the potential usefulness of complementary approaches in CV risk assessment in RA patients.

## 1. Introduction

Cardiovascular diseases (CVD) are a major cause of death in rheumatoid arthritis (RA) patients [1] and clinical events occur approximately 10 years earlier than in the general population [2]. The pooled relative risk of CVD is near 1.5 [3] and cardiac risk is similar to that of type 2 diabetes mellitus (T2DM), especially in RA patients with long disease duration [4].

The CV risk increases in ongoing disease but is already higher than in non-RA subjects at the time of RA diagnosis [5]. The risk seems to be less associated with the traditional (arterial hypertension, diabetes, dyslipidemia, and obesity) or other known CV risk factors (hyperuricemia, hyperhomocysteinemia, and vitamin D deficiency) and more with the disease itself (inflammatory and autoimmune mechanisms) and therapeutic related variables [6]. The relationships with and between each risk factor are complex and have been progressively unraveled in recent years [6].

Considering the increased risk, the EUropean League Against Rheumatism (EULAR) recommends annual CV risk assessment in RA patients (or every 2 to 3 years, if low CV risk and inactive disease occur) [7], with calculation of a modified SCORE index based on traditional CV risk factors, multiplied by 1.5 in the presence of 2 out of 3 RA factors: disease duration > 10 years, certain extra-articular manifestations, or positivity for rheumatoid factor (RF) or anti-cyclic citrullinated peptides (anti-CCP) antibodies.

However, there are uncertainties about the effectiveness of this method in individual CV risk appraisal [5]. In line, several authors have pointed out the need for complementary approaches, including CV imaging techniques and the quantification of newly available biomarkers [8–10].

Myocardial perfusion scintigraphy (MPS) is an imaging method robustly validated in CV risk assessment in several categories of patients [11].

On the other hand, in recent years, new biomarkers that can reflect the interplay between inflammation, atherosclerosis, and plaque instability phenomena have been tested on clinical grounds and some seem promising in CV risk estimation. Serum B-type natriuretic peptides have been clearly associated with CV death and, in RA patients, with CV and general mortality [9]. Less data and conflicting results still exist in what regards the association between another group of mediators, such as osteoprotegerin (OPG) [10], the receptor activator of nuclear factor-kappa B ligand (RANKL) [12], or dickkopf-1 (DKK-1) [13], and the development of CVD in RA.

In this study we included MPS in our risk stratification protocol of patients with established RA. We aimed to evaluate the prevalence of myocardial perfusion defects and to relate the MPS results with various clinical features, including disease characteristics, therapy, traditional CV risk factors, and serum biomarkers [B-type natriuretic peptide (BNP), OPG, RANKL, DKK-1, and sclerostin].

## 2. Patients and Methods

### 2.1. Rheumatoid Arthritis Patients

During a six-month period, patients with RA, classified according to the 1987 American College of Rheumatology criteria [14] and asymptomatic for cardiac disease (angina or heart failure signs and symptoms), were prospectively included in the study protocol. In this time-frame, a total of 216 RA patients were eligible, although only 189 accepted to perform MPS.

The exclusion criteria were history of previous ischemic heart disease, presence of angina, heart failure, or Q waves on basal electrocardiogram (ECG).

Through a rheumatology appointment, all subjects underwent clinical assessment using the Portuguese version of the Stanford Health Assessment Questionnaire (HAQ) and the Disease Activity Score (DAS28) four variables (4v) [15], the most commonly used tools in daily clinical practice to determine the RA disease activity. All data regarding past and ongoing prescribed medications, including NSAIDs and corticosteroids exposure time, were obtained from medical records review. Corticosteroids daily dose was the one at the study inclusion time.

Hypertension, diabetes, and dyslipidemia were defined by self-reporting and contemporary use of compatible medications and/or clinical data assessment in previous medical registries. Office blood pressure (>140/90 mmHg), lipid profile (total cholesterol > 200 mg/dL), and fasting plasma glucose (<126 mg/dL) were categorized according to the European guidelines. The smoking status (past and current) was registered and the body mass index (BMI) calculated.

### 2.2. Laboratory Measurements

Between 08:00 and 10:00 hours in the morning, a fasting blood sample was collected to evaluate the lipid profile [high-density lipoprotein (HDL) cholesterol, low-density lipoprotein (LDL) cholesterol, total cholesterol (TC), triglycerides, lipoprotein (a), apolipoprotein A1 (ApoA1), and apolipoprotein B (ApoB)], as well as glucose and insulin, C-reactive protein (CRP), erythrocyte sedimentation rate (ESR), RF and anti-CCP antibodies, homocysteine, uric acid, 25(OH) vitamin D3, parathyroid hormone, osteocalcin, BNP, OPG, RANKL, DKK-1, and sclerostin levels. Serum samples were stored at −70°C for OPG, RANKL, DKK-1, and sclerostin measurements by ELISA. All these specimens were measured in duplicate, according to the manufacturer's instructions and then averaged. OPG and DKK-1 were determined using kits from BIOMEDICA immunoassays (Vienna, Austria); the intra-assay coefficients of variation (aCV) were ≤3% for both; the inter-aCV was ≤3% for DKK-1 and between 3 and 5% for OPG. RANKL was determined using a kit from CUSABIO (Wuhan, China); the intra-aCV was <8% and the inter-aCV was <10%. Sclerostin was determined using a kit from TECO (Sissach, Switzerland); the intra- and inter-aCVs were 3.8–8.0% and 3.3–9.0%, respectively.

### 2.3. Myocardial Perfusion Scintigraphy

For minimization of exposure to radiation, all patients underwent a routine procedure used in our institution of adenosine-stress single photon emission-computed tomography (SPECT) myocardial perfusion imaging, in a one-day protocol, with the use of stress-first imaging protocol. Images at rest were only acquired if any perfusion defects were detected after the stress phase. This protocol implies the injection of 10 (at stress) to 30 (at rest) mCi doses of TC-99m tetrofosmin, adenosine infused at a rate of 140 *μ*g/kg/min over 6 min, and radiopharmaceutical administration at 3 min of adenosine perfusion.

SPECT imaging was acquired in a Gamma Camera Infinia Hawkeye, GE Healthcare, 15–30 min after radiopharmaceutical administration.

The SPECT images were interpreted in a blinded fashion, by two experienced physicians, consecutively during the study protocol, interspersed with other MPS in patients without RA. Bearing in mind the female predominance [16, 17] attenuation correction (AC) was applied on a case by case basis, depending on patient BMI (≥30 kg/m^2^) and breast volume, using an integrated computed tomography (CT) scanner. A 3 min low-dose CT scan (140 keV, 2.5 mAs) was acquired at the end of stress and rest acquisitions.

Myocardial perfusion was assessed through expert visual analysis in a 17 left ventricle (LV) segmentation model.* QGS/QPS*® software was used to quantify the extension and severity of perfusion defects, wall motion and wall thickening scores, LV end-diastolic and end-systolic volumes (EDV and ESV), and ejection fraction (EF).

Myocardial perfusion was considered abnormal only if the summed stress score (SSS) was ≥4. Perfusion defects were classified as mild (SSS ≥ 4 and ≤8), moderate (SSS > 8 and ≤13), or severe (SSS > 13) and myocardial ischemia was considered significant if the summed difference score (SDS) with respect to MPS at rest was ≥4 [18].

### 2.4. Ethics

Informed consent was obtained from each patient. The study protocol conforms to the ethical guidelines of the 1975 Declaration of Helsinki as reflected in a priori approval (on March 13, 2008) by the institution's human research committee.

### 2.5. Follow-Up Data

All patients were followed during a five-year period. Death and CV events, including the onset of cardiac symptoms, were registered.

### 2.6. Statistical Analyses

Qualitative data are described as absolute counts and proportions and quantitative data as mean (standard deviation). CRP, ESR, and OPG/RANKL ratio were log-transformed to obtain symmetrical distributions. The magnitude of the associations between clinical and laboratory variables and the presence of defects in MPS were assessed using logistic regression models in order to estimate crude odds ratios (OR) and their 95% confidence intervals (CI). All analyses were two-sided and *p* values < 0.05 were considered statistically significant. Since this study was oriented by a priori defined hypotheses no adjustments for multiple comparisons were conducted, as supported by previous literature [19]. Statistical analyses were performed using STATA software V.11 (Statacorp, College Station, Texas, USA).

## 3. Results

### 3.1. Patients Characteristics

One hundred and eighty-nine patients with RA were included, with a mean age of 53 ± 12 years, the majority (*n* = 153, 81%) females. Disease duration, measured from the date of diagnosis, was 14 ± 10 years. The majority of patients presented erosive disease (*n* = 187, 99%) and were seropositive for RF (*n* = 115, 61%) and anti-CCP (*n* = 152, 80%) antibodies. The mean DAS28 (4v) and HAQ were 4.266 ± 1.324 and 1.275 ± 0.708, respectively. DAS28 remission was present in 18 (10%) patients. Extra-articular manifestations were present in 67 (35.4%) patients: Sjögren syndrome (20%), rheumatoid nodes (16%), interstitial lung disease (3%), AA amyloidosis (3%), scleritis (3%), and rheumatoid vasculitis (2%).

Clinical and laboratory variables are shown in Table 1.

### 3.2. Relation of Myocardial Perfusion Scintigraphy Findings with Clinical and Laboratory Parameters

On basal ECG, 184 (97%) patients were in sinus rhythm and 5 (3%) had left bundle branch blockage (LBBB).

MPS showed myocardial perfusion defects (SSS ≥ 4) in 47 (25%) patients, mild defects in 35 (18.5%), moderate in 10 (5.3%), and severe in 2 (1%). The defects had a mean SDS of 4.1 ± 3 and stress extension of 2.5 ± 3.6% and were reversible at rest in 31 (16%) cases. LV ejection fraction (LVEF) was inferior to 45% in 9 (4.76%) patients with a mean value of 63.7 ± 9.8%.

The perfusion defects were either unique (25/47, 53%) or multiple (22/47, 47%), with no preferential distribution at different coronary territories.

**Figure 1:**
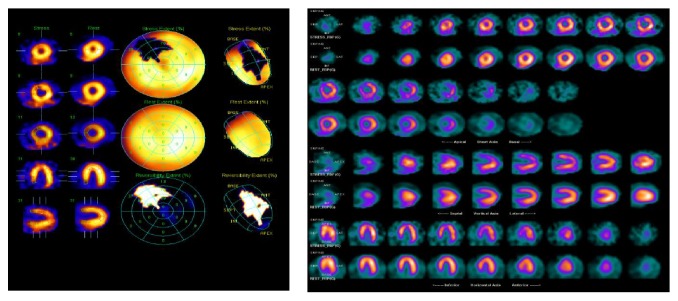
Moderate and reversible anteroseptal perfusion defect compatible with ischemia in the left anterior descending coronary artery territory in an asymptomatic female RA patient.

Figure 1 illustrates an abnormal MPS in female RA patient without any cardiac symptom.

On logistic regression analysis, no relationship was found between the presence of myocardial perfusion defects and patient age, gender, RA disease or therapy related features, smoking habits, history of dyslipidemia, diabetes, or hypertension, neither with determinations, at inclusion protocol, of serum levels of lipids, glucose, or insulin. MPS findings were associated with BMI (OR = 1.120; *p* = 0.002), therapy with ARAs (OR = 2.471; *p* = 0.046), diuretics (OR = 2.195; *p* = 0.045), and oral antidiabetic (OR = 3.031; *p* = 0.033), as well as with circulating levels of BNP ≥ 100 pg/mL (OR = 5.680; *p* = 0.001), DKK-1 ≥ 133 pmol/L (OR = 2.690; *p* = 0.038), and fourth log OPG/RANKL ratio quartile (OR = 2.884; *p* = 0.033) (Table 2).

In a subgroup analysis of those who presented CRP higher than 3 mg/L (upper limit of normality for our laboratory), we found the same associations with similar magnitudes, except for ARAs and diuretics use. MPS findings were associated with BMI (OR = 1.174; *p* = 0.001), oral antidiabetics (OR = 3.840; *p* = 0.017), levels of BNP ≥ 100 pg/mL (OR = 5.610; *p* = 0.004), DKK-1 ≥ 133 pmol/L (OR = 3.142; *p* = 0.056), and fourth log OPG/RANKL ratio quartile (OR = 2.410; *p* = 0.001).

### 3.3. Follow-Up Data: Cardiovascular Events

During a period of 5 years, 9 patients deceased, 2 from CV causes: two sudden cardiac deaths, one of them after an acute myocardial infarction (MI) (confirmed by autopsy). In these two male patients, LVEF was inferior to 45% and both presented perfusion defects on MPS.

Apart from the CV deaths, three other patients developed CV events. A male patient, with LBBB without perfusion defects and with normal LVEF, had MI four years after MPS evaluation. A female patient, with a mild perfusion defect and normal LVEF, developed angina and was submitted to percutaneous stent implantation in the anterior descendent coronary artery. Another female patient, without perfusion defects and with normal LVEF, had a stroke four years after MPS evaluation. All these 5 patients with CV events were seropositive for RF and anti-CCP antibodies, were nonobese, and had low to moderate disease activity by DAS28 and a normal BNP value at baseline and at least one traditional CV risk factor under treatment.

## 4. Discussion

The major finding of this study comprised the description of myocardial perfusion scintigraphy findings in a large RA patient sample, characterized by a long disease duration, with moderate activity, and with no apparent cardiac symptoms. The relationship of MPS results with several clinical variables, including traditional CV risk factors and novel biomarkers of atherosclerosis, was also evaluated.

RA is characterized by earlier atherosclerosis and CV events in comparison with the general population [2, 20]. Overall, the pooled relative risk is 1.68 and 1.87 for myocardial infarction and congestive heart failure (HF), respectively [3]. Myocardial ischemia is often asymptomatic and clinical detection is frequently made at an advanced stage. HF is a major cause of morbidity and mortality in RA, accounting for about 20% of mortality in patients with RA [21]. The risk of HF has been associated with RF positivity, ESR, severe extra-articular involvement, and steroids use [22].

As most CV events are of cardiac origin, it seems appropriate to consider the integration of cardiac imaging in primary prevention strategies, at least in the highest risk patients. However we have few clinical tools able to a priori identify these patients and, until now, few studies have assessed the presence of silent ischemia in large RA patients populations [23].

MPS is a robustly validated risk stratification method in different populations, and a normal MPS is associated with ≤1%/year combined mortality and nonfatal infarction rate [24]. It is a noninvasive functional test that depends not only on the presence of epicardial coronary stenosis (macrovascular disease) but also on the endothelial function, including in the small vessels territories (microvascular disease), which may be early affected during disease development and can be modulated by the inflammatory state.

It is important to note that the nature of the perfusion defects can have an ischemic/atherosclerotic etiology but could also result from other causes like cardiac amyloidosis [8] or interstitial fibrosis. The clinical impact of discriminating the different causes of perfusion defects, for instance, by cardiac magnetic resonance is still unknown.

No relationship was found between the MPS results and the classic CV risk factors. The lack of associations could be explained by the well-known complex interplay between inflammation and therapy that could alter the link between risk factors and atherosclerosis manifestations, as already pointed by other authors [25].

The fact that in our patients we did not find relations between MPS results and the* traditional* risk factors can thus be explained by the fact that the usual relationships were changed by the inflammatory RA disease activity and/or by medications such as with nonbiologic (85%) or biological (51%) DMARDs.

ESR and CRP levels are related to disease activity and inflammation; seropositivity also increases CV risk in RA patients [26]. On the other hand, high CRP levels are related to an increased intima media thickness [27] and baseline values also predict accelerated brachial arterial wall changes in patients with recent-onset RA [28].

In our study, the presence of perfusion defects on MPS was not related to systemic inflammatory markers (CRP and ESR), nor with the presence of RF and anti-CCP antibodies or DAS28 score.

The inflammatory state could have changed over time, especially under therapy, and, later in disease progression, it could be difficult to relate a unique plasma level (mean CRP value of 11.57 ± 20.0 mg/L in our patients) with the extension of a long lasting process of cardiac damage.

B-type natriuretic peptides (BNP and NT-proBNP) are independent predictors of CV morbidity and of all causes of mortality including in RA patients.

In early inflammatory polyarthritis, NT-proBNP has been associated with HAQ score and CRP [29] and this fact may be indicative of the link between joint and cardiac (myocardial and/or vascular) structural damage induced by chronic inflammatory diseases.

In our RA patients, with a longer disease duration, BNP was poorly correlated with HAQ (Spearman correlation; *r* = 0.189, *p* = 0.007), but levels superior to 100 pg/mL were strongly associated with the presence of perfusion defects on MPS independently of LV EF.

This result corroborates the hypothesis that MPS could reveal subclinical cardiac dysfunction and supports the utility of BNP measurement as a screening tool in the assessment of CV risk in RA patients. This hypothesis needs to be corroborated with further studies.

OPG and RANKL are bone-controlling cytokines of mineral metabolism but also participate in the process of atherogenesis, calcification, and plaque rupture at the vascular walls [30].

In vascular atherogenic remodeling process, OPG could be increased due to a secondary compensatory mechanism activation [31], and higher OPG serum levels have been associated with coronary atherosclerosis [32], silent myocardial ischemia, increased CV mortality [33], myocardial infarction, and heart failure prognosis [34].

By contrast, RANKL promotes calcification at the vascular wall and it has been mostly linked with the risk of plaque instability and rupture [35].

In our study, we did not find associations between myocardial perfusion defects with either RANKL or OPG isolated levels, but rather with the highest quartile of the distribution of the OPG/RANKL ratio. This elevated ratio may be due to the presence of an ongoing vascular atherosclerosis (high OPG) in a still stable and low-risk plaques (low RANKL) in patients with abnormal MPS.

DKK-1 and sclerostin, two Wnt pathway inhibitors, are also involved in the atherosclerosis process. DKK-1 is linked not only with the effects of TNF*α* on joints in RA [36] but also with platelet-mediated endothelial cell activation [13], endothelial dysfunction in T2DM [37], vascular calcification [38], and premature myocardial infarction [39]. DKK-1 serum concentration has already been correlated with the presence of coronary artery calcification and atherosclerosis [40], and its measurement has been proposed as a simple test that might be useful in CV risk stratification. Our results corroborate this last hypothesis since we found a significant association of DKK-1 levels with the presence of myocardial perfusion defects.

The occurrence of few number of CV events in our patients precludes statistical analysis for predictor variables but significant reductions of cardiovascular events have been reported with the use of biological compared with conventional DMARDs in RA patients [41–43].

## 5. Study Limitations

The major limitation of this study is related with AC in MPS which was not performed in all examinations and this might have contributed to overestimating the prevalence of perfusion defects in some patients; however, AC might also introduce false-positive perfusion defects particularly among the female population.

On the other hand, there was no coronary anatomy control that can guarantee the atherosclerotic nature of all perfusion defects nor the presence of balanced ischemia.

The low representativeness of male and early RA patients precludes the extrapolation of obtained results to both genders and to patients with less disease duration.

## 6. Conclusions

A significant proportion of asymptomatic RA patients presented myocardial perfusion abnormalities in MPS. The association with BNP, OPG/RANKL ratio, and DKK-1 levels put in perspective the use of complementary approaches, including serum biomarkers quantification in CV risk stratification. Our results corroborated the hypothesis that MPS could reveal subclinical cardiac dysfunction and supported the utility of BNP measurement as a screening tool in CV risk assessment of RA patients.

More studies are needed to definitely evaluate the utility of different strategies (imaging, biomarkers) in the clinical setting, particularly in the era of increasing availability of RA biological therapies.

## Figures and Tables

**Table 1:** Clinical and laboratory variables of the rheumatoid arthritis sample enrolled in the study.

Characteristic	Value
*Patient related*	
Female, *n* (%)	153 (81.0)
Postmenopausal, *n* (%)	59 (31.2)
Time since menopause, mean (SD), years	7.330 (8.275)
Age, mean (SD), years	53.340 (11.502)
BMI, mean (SD), kg/m^2^	27.150 (4.768)
Ever smokers, *n* (%)	24 (12.7)
Hypertension, *n* (%)	39 (20.6)
Diabetes mellitus, *n* (%)	14 (7.4)
Dyslipidemia, *n* (%)	23 (12.2)
*Disease related*	
Disease duration, mean (SD), years	13.72 (9.955)
RF positivity, *n* (%)	115 (60.847)
Anti-CCP antibodies positivity, *n* (%)	152 (80.423)
DAS28 (4V), mean (SD)	4.266 (1.324)
Remission (DAS28 (4v) < 2.6)	18 (9.5)
Low (2.6 < DAS28 (4v) ≤ 3.2)	27 (14.3)
Moderate (3.2 < DAS28 (4V) ≤ 5.1)	95 (50.3)
High (DAS28 (4v) > 5.1)	49 (25.9)
ESR, mean (SD), mm (first hour)	27.100 (20.603)
CRP, mean (SD), mg/L	11.573 (20.045)
Global health on VAS, mean (SD)	44.190 (17.794)
HAQ score, mean (SD)	1.275 (0.708)
*RA related treatment *	
Corticosteroids, *n* (%)	155 (82.0)
Daily dose in prednisolone equivalents, mean (SD), mg	5.139 (3.977)
Time under corticosteroids, mean (SD), years	11.429 (8.981)
NSAIDs, *n* (%)	142 (75.1)
DMARDS, *n* (%)	160 (84.7)
Cumulative, mean (SD)	2.534 (1.416)
Time under DMARDs, mean (SD), months	104.037 (87.714)
Methotrexate, *n* (%)	130 (68.8)
Leflunomide, *n* (%)	40 (21.1)
Biologic DMARDs, *n* (%)	97 (51.3)
Time under biologics, mean (SD), months	60.258 (30.080)
Anti-TNF*α* blockers, *n* (%)	83 (43.9)
Non-anti-TNF*α* blockers, *n* (%)	14 (7.4)
Bisphosphonates, *n* (%)	69 (36.5)
*Cardiovascular related treatment*	
Antiaggregants, *n* (%)	22 (11.6)
ACEIs, *n* (%)	24 (12.7)
ARAs, *n* (%)	24 (12.7)
CCAs, *n* (%)	18 (9.5)
BBs, *n* (%)	15 (7.9)
Diuretics, *n* (%)	37 (19.6)
Warfarin, *n* (%)	5 (2.6)
Statins, *n* (%)	45 (23.8)
Oral antidiabetic agents, *n* (%)	17 (9.0)
Insulin, *n* (%)	5 (2.6)

ACEIs, angiotensin converter enzyme inhibitors; ARAs, angiotensin receptor antagonists; BBs, beta-blockers; BMI, body mass index; CCAs, calcium channel antagonists; CCP, cyclic citrullinated peptides; CRP, c-reactive protein; DAS, disease activity score; DMARDS, disease-modifying antirheumatic dugs; ESR, erythrocyte sedimentation rate; HAQ, health assessment questionnaire; NSAIDs, nonsteroidal anti-inflammatory drugs; RF, rheumatoid factor; SD, standard deviation; TNF*α*, tumour necrosis factor alpha; VAS, visual analogic scale.

**Table 2:** Crude odds ratios for the associations between rheumatoid arthritis patients' characteristics and the presence of myocardial perfusion defects obtained by logistic regression analysis.

		95% CI for OR		
	OR	Lower	Upper	*p* value
Male sex (versus female)	1.009	0.436	2.334	0.984
Age (per year)	1.000	0.971	1.029	0.989
Disease duration (per year)	1.013	0.981	1.047	0.435
*BMI (per kg/m* ^*2*^)	*1.120*	*1.044*	*1.202*	*0.002*
Ever smoker (versus never smoker)	1.008	0.375	2.711	0.987
Smoking exposure (per year)	1.002	0.918	1.093	0.971
Time under biologics	1.002	0.993	1.010	0.729
DAS28 (4v) (per unit)	0.913	0.710	1.176	0.482
HAQ (per unit)	0.930	0.582	1.485	0.760
log CRP (per unit)	1.129	0.872	1.464	0.357
log ESR (per unit)	0.896	0.562	1.428	0.644
*BNP (pg/mL)*				
<30	Reference	Reference	Reference	Reference
≥30 and <100	1.635	0.780	3.428	0.193
*≥100*	*5.680*	*2.038*	*15.830*	*0.001*
Insulin resistance				
<1	Reference	Reference	Reference	Reference
≥1 and <1.5	2.260	0.816	6.263	0.117
≥1.5 and <2.5	1.361	0.500	3.707	0.546
≥2.5	1.005	0.322	3.135	0.994
TC/HDL ratio (per unit)	0.987	0.704	1.386	0.941
LDL/HDL ratio (per unit)	0.980	0.643	1.495	0.927
ApoB/ApoA1 ratio (per unit)	0.400	0.066	2.424	0.319
TC (per mg/dL)	0.935	0.368	2.377	0.888
LDL (per mg/dL)	0.952	0.295	3.070	0.934
HDL (per mg/dL)	1.005	0.103	9.801	0.996
TGs (per mg/dL)	1.338	0.774	2.314	0.297
ApoA1 (per mg/dL)	1.004	0.992	1.016	0.508
ApoB (per mg/dL)	0.995	0.980	1.010	0.526
Lp(a) (mg/dL) (per unit)	0.985	0.969	1.002	0.076
Uric acid (per mg/L)	1.010	0.989	1.032	0.359
Homocysteine (per *µ*mol/L)	1.013	0.931	1.103	0.757
25(OH) vitamin D3 (per ng/mL)	1.023	0.998	1.048	0.077
Osteocalcin (per ng/mL)	1.010	0.968	1.054	0.651
*DKK-1 (pmol/L) *(per unit)	*1.010*	*1.002*	*1.017*	*0.010*
<60	Reference	Reference	Reference	Reference
≥60 and <90	0.778	0.263	2.297	0.649
≥90 and <133	1.657	0.630	4.355	0.306
*≥133*	*2.690*	*1.058*	*6.840*	*0.038*
Sclerostin (per ng/mL)	2.071	0.063	68.436	0.683
*log OPG/RANKL (per unit)*	*1.667*	*1.106*	*2.513*	*0.015*
First quartile	Reference	Reference	Reference	Reference
Second quartile	1.357	0.481	3.828	0.564
Third quartile	1.537	0.553	4.268	0.410
*Fourth quartile *	*2.884*	*1.091*	*7.622*	*0.033*
Osteoprotegerin (per pmol/L)	1.161	0.987	1.366	0.072
RANKL (per pmol/L)	0.999	0.999	1.000	0.111
Prednisolone (mg a day) (per unit)	1.066	0.984	1.154	0.119
Corticosteroid therapy duration (years) (per unit)	1.010	0.974	1.047	0.588
Methotrexate (mg once a week) (per unit)	0.992	0.952	1.034	0.713
NSAIDs use (current versus not current)	0.823	0.391	1.737	0.610
Bisphosphonates use (ever versus never)	0.671	0.330	1.365	0.271
ACEIs use (current versus not current)	1.615	0.643	4.060	0.308
*ARAs use (current *versus *not current)*	*2.471*	*1.015*	*6.018*	*0.046*
CCAs use (current versus not current)	1.585	0.560	4.490	0.386
BBs use (current versus not current)	1.108	0.335	3.660	0.867
*Diuretics use (current *versus *not current)*	*2.195*	*1.018*	*4.732*	*0.045*
Antidyslipidemic therapy (current versus not current)	0.934	0.430	2.029	0.863
Insulin therapy (current versus not current)	2.059	0.333	12.715	0.437
*Oral antidiabetic therapy (current *versus *not current)*	*3.031*	*1.096*	*8.382*	*0.033*
Diabetes mellitus	1.759	0.559	5.539	0.334
Arterial hypertension	1.460	0.671	3.180	0.340

ACEIs, angiotensin converter enzyme inhibitors; Apo, apolipoprotein; ARAs, angiotensin receptor antagonists; BBs, beta-blockers; BMI, body mass index; BNP, brain natriuretic peptide; CCAs, calcium channel antagonists; CI, confidence interval; CRP, c-reactive protein; ESR, erythrocyte sedimentation rate; DAS, disease activity score; DKK-1, dickkopf-1; HAQ, health assessment questionnaire; HDL, high-density lipoprotein; LDL, low-density lipoprotein; Lp(a), lipoprotein (a); NSAIDs, nonsteroidal anti-inflammatory drugs; OPG, osteoprotegerin; OR, odds ratio; RANKL, receptor activator of nuclear factor-kappa B ligand; TC, total cholesterol; TGs, triglycerides.
